# Comprehensive analysis of kampo medicine utilization across all outpatient departments with a focus on anesthesiology department/pain clinic at Kyoto university hospital over 12 years

**DOI:** 10.1186/s40780-025-00486-7

**Published:** 2025-08-23

**Authors:** Karin Kato, Kaori Tsuyuki, Takuma Ohsuga, Akihiko Ueda, Miho Egawa, Masashi Ikuno, Neiko Ozasa, Kiyoaki Tanikawa

**Affiliations:** 1 Kampo medical unit, Kyoto University Hospital, 54 Shogoin-kawahara-cho, Sakyo-ku, Kyoto City, 606-8507 Kyoto Japan; 2 Department of Patient Safety Kyoto University Hospital, Kyoto City, Japan; 3 Department of Gynecology and Obstetrics, Kyoto University Graduate School of Medicine, Kyoto City, Japan; 4 Department of Biomedical Data Intelligence, Graduate School of Medicine, Kyoto University, Kyoto City, Japan; 5 Center For Medical Education and Internationalization, Kyoto University, Kyoto City, Japan; 6 Department of Rehabilitation, Department of Cardiology, Kansai Heart Center, Takanohara Central Hospital, Takanohara City, Japan; 7 Tanikawa Clinic, Toyama City, Japan

**Keywords:** Kampo, Medication, Pain clinic/Anesthesiology, Survey, Traditional

## Abstract

**Aim:**

Kampo medicines, which are covered under national health insurance, are widely accessible and affordable in Japan; however, knowledge about their current prescription practices remains limited. This study investigated the outpatient prescription practices of Kampo formulations at Kyoto University Hospital from April 2011 to March 2023.

**Results:**

Kampo medicine was prescribed in all medical departments. A total of 42,453 prescriptions were recorded during the study period, with an average of 3,538 prescriptions per year. The gynecology and obstetrics department had the highest number of Kampo prescriptions, followed by anesthesiology. The anesthesiology department prescribed various formulations for 12 years. The usage rate of Processed Aconite Root-containing formulations increased with increasing patient age, reaching 3.3% in individuals in their teens, 6.6% in their twenties, 13.3% in their thirties, and over 30% in their eighties and nineties.

**Conclusion:**

The substantial use of Kampo medicines across various departments indicates their critical role in specialized and general medical practice. The adaptability and personalized approach of treatments using Kampo medicines in the anesthesiology department are evident from the dynamic prescription patterns. Notably, the increasing use of Processed Aconite Root-containing formulations with advancing patient age suggests a potential age-related preference or therapeutic indication. These findings underscore the integral role of Kampo medicine in modern clinical practice and emphasize the necessity of further research into its age-specific applications and departmental prescribing trends.

## Introduction

Kampo medicine, Japan’s traditional herbal medical system, originated from classical Chinese medicine and was introduced to Japan via the Korean Peninsula during the 5th to 6th centuries. A pivotal moment occurred in 742 AD when the Tang dynasty monk Jian Zhen (Ganjin) brought Chinese medicinal texts and crude drugs to Japan, laying the foundation for Kampo practice [1–3]. Over time, Kampo evolved independently, adapting to Japan’s climate, culture, and patient constitution, while classical Chinese texts helped systematize its diagnostic and therapeutic principles [4].

In 1976, Kampo formulations were officially included under Japan’s National Health Insurance system, marking their formal integration into modern medical practice. This inclusion made Kampo medicines accessible to a wide range of patients at a relatively reasonable cost [5]. Today, Kampo medicine is regulated by the Ministry of Health, Labour and Welfare and is widely used in clinical settings [6]. Over 80% of Japanese physicians incorporate Kampo into their practice, frequently in combination with Western medicine, reflecting its complementary role in clinical care. Kampo medicines are available both as prescription drugs and over-the-counter products (OTC) [7], and are dispensed in hospitals, clinics, and dedicated Kampo outpatient departments [8–10]. A wide variety of Kampo formulations enables the provision of personalized treatment tailored to individual patient needs and conditions [11]. These characteristics demonstrate the integral role of Kampo medicines in Japan’s healthcare system. Furthermore, evidence-based research on Kampo’s efficacy and safety continues to expand, reinforcing its role in contemporary healthcare [12].

Internationally, the World Health Organization has acknowledged Kampo within the International Classification of Diseases, 11th Revision (ICD-11), Chap. 26, which includes traditional medicine conditions from Chinese, Japanese, and Korean systems [13, 14].

Kyoto University Hospital, which is renowned for its comprehensive medical services, extensive research programs, and interdisciplinary care, is affiliated with Kyoto University, one of Japan’s most prestigious universities. The Kampo medical unit was established in April 2016 at Kyoto University and serves as a cross-sectional organization within the affiliated hospital, specializing in Kampo medicine. It has received the International Organization for Standardization (ISO) 9001 management system certification, reflecting its adherence to international quality management standards [15].

A wide range of departments and divisions, including anesthesiology, neurology, obstetrics and gynecology, cardiology, hepatobiliary transplant surgery, plastic surgery, breast surgery, pharmacy, and orthopedic surgery, is included in the Kampo medical unit. The unit’s outpatient clinic caters to patients who experience side effects from Western treatments and those whose symptoms are not adequately managed using Western medicine alone.

The anesthesiology department is a core member of the Kampo medical unit. In this unit, physicians explain the best treatment options to patients and seek their preferences. Moreover, they decide whether Kampo medicine alone or in combination with Western medicine is more suitable for a particular patient. Several physicians work together as a team to provide treatments using Kampo medicines. This unit, emphasizing interdepartmental cooperation, allows doctors from various specialties to exchange opinions and collaborate on patient care, thereby ensuring the provision of comprehensive and integrated treatment.

The Anesthesiology Outpatient Clinic, also known as the Pain Clinic, primarily treats patients experiencing pain in various parts of their bodies. In Western medicine, the range of pain relief medications is limited. In Japan, narcotics require special certification in addition to a medical license, and their prolonged use is not permitted for providing pain relief to patients other than those with cancer. Due to these limitations, the anesthesiology department frequently prescribes Kampo medicines consisting of Processed Aconite Root (as defined in the Japanese Pharmacopoeia, 18th Edition) [16, 17]. Therefore, anesthesiologists have been considered the prime members of the Kampo medical unit.

Until 2019, the anesthesiology department had five weekly consultation slots. However, these slots were reduced due to global medical restrictions following the onset of the COVID-19 pandemic in 2020. In 2021, the resignation of a specialist in Kampo medicines resulted in the closure of their outpatient slots, thereby further reducing the consultation slots. Therefore, the nature of outpatient care was considered to differ across three time periods: 2011–2015, prior to the establishment of the Kampo medical unit; 2016–2019, after the launch of the Kampo medical unit; and 2020–2022, during the reduction of outpatient slots due to the COVID-19 pandemic.

In this study, a survey was conducted to better understand the current state of Kampo prescriptions and enhance future treatments with Kampo medicines in accordance with the patient needs. This study specifically investigated the usage of Kampo extract formulations and examined the differences in prescription practices across various departments based on actual data. In total, 44 Kampo extract formulations are available for prescription in Kyoto University Hospital during the 12-year period.

## Methods

### Design

During the study period, prescription data of Kampo extract formulations and ethical crude drug products were extracted from the electronic medical records at Kyoto University Hospital. Only Kampo formulations that were consistently adopted by our hospital throughout the 12-year study period were included. Formulations that were introduced mid-way or discontinued during the study period were excluded from the analysis. In this study, the term “prescription count” refers to the number of times an individual Kampo pharmaceutical product was prescribed, regardless of dosage or duration. We count repeated prescriptions of the same Kampo formulation for the same patient within the same fiscal year as a single prescription event. We also extracted the number of days prescribed in a single prescription event. We included only data on regular prescriptions and excluded data on as-needed prescriptions. The prescriptions were analyzed based on department, age groups (in 10-year increments), year, and specific ingredients. In addition, the total number of outpatient visits for each year was determined. The prescriptions were categorized in three time periods: 2011–2015, before the establishment of the Kampo medical unit; 2016–2019, after the launch of the Kampo medical unit; and 2020–2022, during the reduction of outpatient slots due to the COVID-19 pandemic. The frequent prescriptions issued by the anesthesiology department in each period were examined.

### Settings

The Kyoto University Hospital Ethics Committee (R3806) approved this single-center, retrospective study. The study was conducted in accordance with the principles of the Declaration of Helsinki and the Ethical Guidelines for Medical and Health Research Involving Human Subjects issued by the Ministry of Health, Labor and Welfare. Patients who visited Kyoto University Hospital between April 2011 and March 2023 and were prescribed Kampo extract formulations were included in this study. To maintain consistency and avoid interpretive complexity in longitudinal trend analysis, our analysis was based on Kampo formulations continuously adopted at our hospital over the 12-year period. Including them would have required adjusting for variable availability, which could compromise the clarity and comparability of the results.

### Statistical analysis

Categorical variables are presented as numbers and percentages. The average, maximum, and minimum values of the data were calculated using Excel functions. Microsoft Excel was used for calculating the coefficient of determination (R²) to analyze the trend of the data and determine the degree of correlation. A linear trending was applied to the scatter plot. The R² value provided a quantitative measure of how well the data fitted the linear regression model, indicating the proportion of variance in the dependent variable that could be explained by the independent variable.

In addition, the proportion of patients that were prescribed formulations containing Processed Aconite Root (hachimijiogan, keishikajutsubuto, shimbuto, daibofuto, goshajinkigan, and maobushisaishinto, based on the package inserts) were investigated based on age groups (teens, 20s, 30s, etc.).

## Results

Over the 12-year study period, Kampo extract formulations were prescribed by the following departments and divisions: hematology, endocrinology, diabetes and nutrition, cardiology, gastroenterology, pulmonology, immunology and rheumatology, general medicine and emergency care, neurology, nephrology, oncology, gastrointestinal surgery, breast surgery, hepatobiliary pancreatic and transplant surgery, pediatric surgery, cardiovascular surgery, thoracic surgery, neurosurgery, ophthalmology, otorhinolaryngology, head and neck surgery, dentistry and oral surgery, orthopedic surgery, plastic surgery, obstetrics and gynecology, urology, anesthesiology, pediatrics, radiology, dermatology, psychiatry, geriatric medical unit, Kampo medical unit, and rheumatology center.

**Fig. 1:**
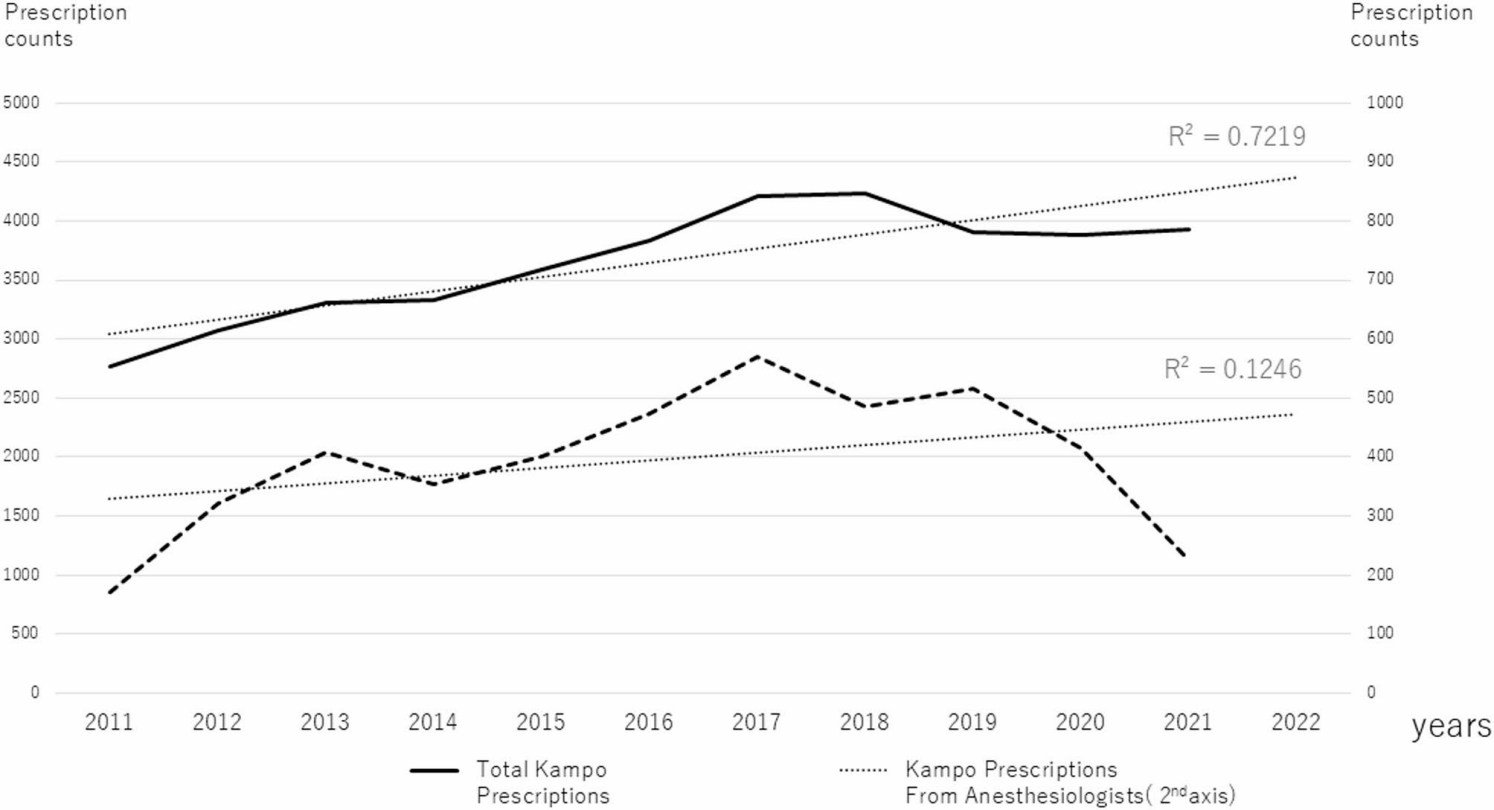
Annual Kampo prescription counts across all departments (black line) and anesthesiology department (dotted line). The horizontal axis represents the fiscal years. The primary Y-axis (on the left) indicates prescription counts. The dotted line is plotted against the secondary Y-axis (on the right)

Over this period, 42,453 Kampo extract formulation prescriptions events were issued by various departments in the hospital. The average annual number of prescriptions was 3,538. The lowest number of prescriptions issued was 2,394 in 2011 and the highest was 4,231 in 2018. In total, 4,482 Kampo extract formulation prescriptions were issued specifically by the anesthesiology department, with an average of 373 prescriptions annually. The lowest number of prescriptions issued by the anesthesiology department was 139 in 2011 and the highest was 569 in 2018. Figure 1 shows the trends in Kampo prescriptions in all medical departments and anesthesiology departments over a 12-year period. The overall number of prescriptions issued at Kyoto University Hospital revealed a moderate positive correlation (R² = 0.722) between the year and the annual number of Kampo prescription records over the 12-year period from 2011 to 2022. The number of Kampo prescriptions by anesthesiologists has been on the decline since around 2020.

**Fig. 2:**
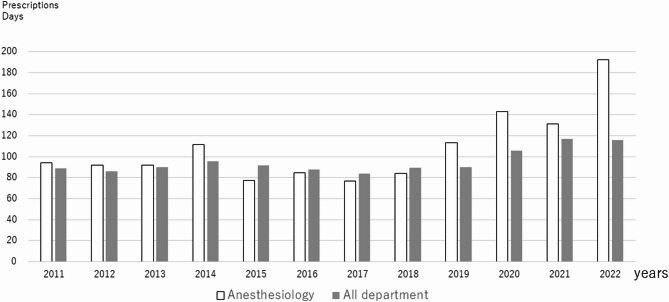
Average number of prescription days of Kampo medicine. The white bar graph shows prescriptions by anesthesiologists, and the gray bar graph shows prescriptions by all medical departments. The horizontal axis represents the fiscal years

Figure 2 shows the average Kampo prescriptions days in all medical departments and anesthesiology departments over a 12-year period. The number prescriptions days for all medical departments has remained at around 90 days, while the number prescription days by anesthesiologists has been increasing since around 2020.

Table 1 lists the departments that issued the highest number of prescriptions over the 12-year period. The obstetrics and gynecology department issued the highest number of prescriptions, followed by anesthesiology; neurology; pulmonology; endocrinology, diabetes, and metabolism; psychiatry; rheumatology and clinical immunology; cardiology; orthopedic surgery; and gastroenterology.

**Table 1:** The departments with the highest number of Kampo prescriptions issued over 12 years

Department	*n*(prescriptions)
Obstetrics and Gynecology	5435
Anesthesiology	4482
Neurology	3824
Pulmonology	3071
Endocrinology, Diabetes, and Nutrition	2995
Psychiatry	2729
Rheumatology and clinical immunology	1902
Cardiology	1895
Orthopedic Surgery	1608
Gastroenterology	1538

**Fig. 3:**
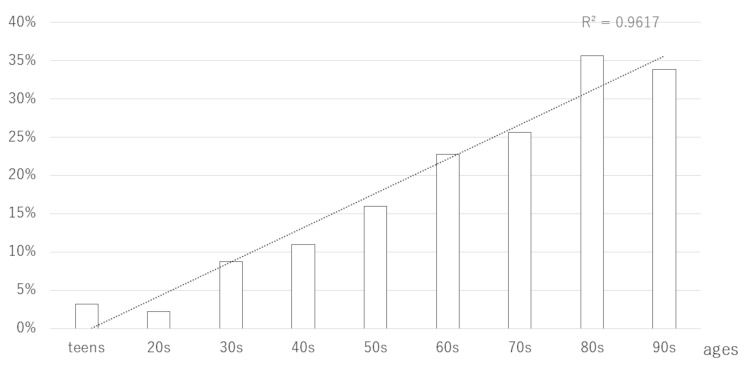
Proportion of prescriptions containing Processed Aconite Root given by the anesthesiology department based on the year. The horizontal axis represents age groups (teens, 20s, 30s, etc.). The black bar graph represents the proportion of formulations containing Processed Aconite Root (hachimijiogan, keishikajutsubuto, shimbuto, daibofuto, goshajinkigan, and maobushisaishinto) prescribed by the anesthesiologists. The dotted line represents the trend line. Annual number of Kampo prescription events and linear regression analysis. The R² value (0.757) indicates the strength of the correlation between year and prescription events

Figure 3 depicts the proportion of prescriptions containing Processed Aconite Root issued by the anesthesiology department with respect to the ages of the patients. The prescription of formulations containing Processed Aconite Root increased with patient age, with the recipients including 3.2% of patients in their teens, 2.2% in their twenties, 8.8% in their thirties, and over 30% in their eighties and nineties.

Table 2 demonstrates the frequent prescriptions issued by the anesthesiology department during the following three periods: 2011–2015 (before the establishment of the Kampo medical unit); 2016–2019 (after the launch of the Kampo medical unit); and 2020–2022 (during the reduction of outpatient slots due to the impact of the COVID-19 pandemic). The most frequently prescribed Kampo formulations before the establishment of the Kampo medical unit 2015 were goreisan (10.2%), keishikajyuutubuto (9.1%), kakkonto (8.1%), keishibukuryogan (8.0%), and yokukansan (7.1%). The most common prescriptions prescribed from 2016 to 2019 were keishibukuryogan 8.1%), Processed Aconite Root (6.4%), gosyajinkigan (6.2%), goreisan (6.0%), and hochuekkito (4.7%). Post 2020, the most frequently prescribed formulations were sokeikakketuto (9.7%), keishibukuryogan (8.5%), goreisan (7.4%), Processed Aconite Root (6.9%), and hochuekkito (6.0%).

**Table 2:** The highest total number of prescriptions issued over 12 years by the anesthesiology department, divided in three periods: 2011–2015 ,prior to the establishment of the Kampo medical unit; 2016–2019, following the launch of the Kampo medical unit; and 2020–2022, during the period of reduced outpatient slots due to the impact of the COVID-19 pandemic

2011–2015 (year) Before Kampo Unit		2016–2019 After Kampo Unit		2020–2022 After Covid19	
Prescription	(%)	Prescription	(%)	Prescription	(%)
Goreisan	10.2	Keishibukuryogan	8.1	Sokeikakketuto	9.7
Keishikajyutubuto	9.1	Processed Aconite Root	6.4	Keishibukuryogan	8.5
Kakkonto	8.1	Gosyajinkigan	6.2	Goreisan	7.4
Keishibukuryogan	8.0	Goreisan	6.0	Processed Aconite Root	6.9
Yokukansan	7.1	Hochuekkito	4.7	Hochuekkito	6.0
Kamisyoyosan	6.9	Syakuyakukanzoto	4.3	Syakuyakukanzoto	4.1
Syakuyakukanzoto	6.2	Kamisyoyosan	4.2	Goshajinkigan	4.0
Goshajinkigan	5.3	Kakkonto	4.2	Juzentaihoto	4.0
Hangekobokuto	5.0	Rikkunshito	4.0	Kakkonto	3.3
Tokisyakuyakusan	4.8	Keishikazyutubuto	4.0	Keishikazyutubuto	3.3

## Discussion

Kampo medicines hold a unique position in the Japanese healthcare system, characterized by their integration into the standard medical practice [6, 10]. They can be prescribed by all licensed physicians, and several Kampo medicines are accessible and affordable as they are covered under the national health insurance. The wide array of available formulations, supported by scientific research, enables the provision of personalized treatment tailored to individual needs. The findings of this study reflect these distinctive features and highlight the extensive use and acceptance of Kampo medicines across various medical departments. Such integration underscores their importance and effectiveness within the Japanese healthcare framework.

This study explored the prescription patterns of Kampo extract formulations over a 12-year period at a university hospital having an interdisciplinary Kampo medical unit, with a focus on the anesthesiology department. Initially, the Kampo extract formulations were confirmed to be prescribed by all clinical departments and treatment divisions. Kampo extract is routinely used in all clinical departments at Kyoto University Hospital. The substantial number of prescriptions highlights the need for thoroughly examining these prescriptions. This widespread use underscores the importance of understanding Kampo medicines and their potential side effects by all medical practitioners [18]. Given their extensive utilization across various departments, Kampo medicines are not only essential for specialized care but also for general medical practice.

The top three departments with the highest number of Kampo prescriptions belonged to the Kampo medical unit. The department the issued the second highest number of Kampo medicine prescriptions at our hospital was the anesthesiology department. Traditionally, Kampo medicines have been frequently prescribed in various departments, such as obstetrics and gynecology, fertility clinics, internal medicine, and for postsurgical use [6]. These results highlight a unique characteristic of Kyoto University Hospital. The high number of Kampo prescriptions issued by anesthesiologists at our hospital can be attributed to several factors. First, anesthesiologists are the central members of the Kampo medical unit. In addition, the only department to have a full-time Kampo specialist among the other attending physicians is the anesthesiology department. The presence of the specialist ensures that Kampo prescriptions are well-balanced and are not overly composed of any single formulation. The diversity in the issued prescriptions reflects that the treatments are tailored to each patient’s specific condition rather than relying on standardized or popular remedies.

The high number of Kampo prescriptions in obstetrics and gynecology reflects the department’s management of diverse conditions across women’s life stages, including postoperative intestinal dysfunction, menopausal disorders, menstrual disorders, and pregnancy-related minor complications, all of which are well-suited to Kampo therapy [19–21]. A detailed analysis of this trend will be conducted in a future study.

A comparison of the prescription patterns of Kampo medicines across different departments can highlight specific clinical scenarios or patient populations where these medications are particularly beneficial. It can also reveal any disparities in prescribing practices that might require further investigations.

Therefore, this study focused on the anesthesiology department, which plays a central role in the Kampo medical unit at Kyoto University Hospital. The extensive use of Kampo medicines in the anesthesiology department is particularly noteworthy, with the department ranking second in Kampo prescriptions over the 12-year period. Due to the reduction in outpatient slots following the outbreak of the COVID-19 pandemic, the total number of Kampo prescriptions decreased. Although the number of prescriptions for Kampo medicine has decreased, the number of prescription days has increased, suggesting that the number of patients taking Kampo medicine has not decreased.

As depicted in Table 2, the most prescribed Kampo formulations by anesthesiologists have varied throughout the years. This variation could be attributed to the fact that the anesthesiology department treats patients with diverse types of pain affecting various parts of the body, and anesthesiologists tailor their prescriptions considering each patient’s specific symptoms instead of prescribing treatments for a single typical condition [10]. Although the drugs were ranked high in prescriptions, there were formulas included that had no indications related to “pain”. Formulations such as yokukansan, kamishoyosan, and hangekobokuto (Kampo formulas listed in the Japanese Pharmacopoeia [16]), which are used to address *qi imbalances*[TM: Traditional Medicine], were frequently prescribed between 2011 and 2015. The increased use of yokukansan during this period may be attributed to studies demonstrating its efficacy for behavioral and psychological symptoms of dementia (BPSD), which supported its clinical application and insurance coverage [22–24]. Similarly, kamishoyosan gained attention following studies, which reported its effectiveness in reducing hot flashes and modulating cytokine levels in menopausal women [19, 20]. These publications likely contributed to the rise in prescriptions of these Kampo formulations during the early 2010s. Goreisan and goshajinkigan (Kampo formulas listed in the Japanese Pharmacopoeia [16], which are used to treat water metabolism abnormalities, were frequently prescribed from 2011 to 2019. Processed Aconite Root and sokeikakketsuto have become increasingly favored prescriptions since 2016. While keishibukuryogan has been consistently prescribed, the increasing preference for sokeikakketsuto has resulted in declining rankings of keishibukuryogan. Sokeikakketsuto is currently the go-to long-term prescription for static *blood* [TM] and “*Hisho*” [TM] treatments [25]. “*Hisho*” [TM] is a traditional Kampo medicine concept. According to the classical text “Huangdi’s Internal Classic“ [26], the invasion of three pathogenic factors, i.e., *wind*, *cold*, and d*ampness* [TM], or their combinations results in the condition known as “*Hisho*” [TM]. These external pathogenic factors disrupt the normal circulation of *qi* [TM] and *blood* [TM] by entering through the skin, muscles, and joints and invading the meridians. The symptoms of “*Hisho*” [TM] include joint pain, restricted joint movement, numbness, and a heavy, aching sensation in the lower back and knees. Chronic cases can lead to joint deformities, such as those observed in rheumatoid arthritis and knee osteoarthritis. When *wind* [TM] is the primary cause, pain tends to move, is widespread, and is often accompanied with numbness. In such cases, *blood*-activating and nourishing crude drugs, like sokeikakketsuto, are used. When dampness is the main cause, the symptoms include a heavy, aching sensation in the body and limbs, difficulty in movement, and numbness of the skin. When *wind* [TM] is considered the primary factor, formulations such as sokeikakketsuto—containing crude drugs like *Angelica Acutilobae Radix* (the root of *Angelica acutiloba*) and *Paeoniae Radix* (the root of *Paeonia lactiflora*)—are used to activate *blood* [TM] and nourish the body [27]. For dampness-related symptoms, formulations like goreisan (including *Alismatis Rhizoma* [the tuber of *Alisma orientale*] and *Polyporus* [the sclerotium of *Polyporus umbellatus*]) and rikkunshito (including *Ginseng Radix* [the root of *Panax ginseng*] and *Atractylodis Macrocephalae Rhizoma* [the rhizome of *Atractylodes macrocephala*]) are preferred to strengthen the *spleen* [TM] and dispel dampness [28, 29]. In cases where *cold* [TM] is the dominant factor, warming crude drugs such as Cinnamon Bark or Processed Aconite Root are prescribed [30]. These prescriptions reflect the underlying pain clinic principles.

Previous studies have demonstrated that the frequently prescribed Kampo formulations in Japan include shakuyakukanzoto, daikenchuto, and yokukansan [21, 31]. Processed Aconite Root is also one of the most commonly prescribed crude drugs at the anesthesiology department of our hospital. Processed Aconite Root [16] is derived from the tuberous roots of the plants *Aconitum carmichaelii* and *Aconitum japonicum*, which belong to the Ranunculaceae family. The roots are subjected to detoxification before being dried. In Kampo medicines, aconite is known for its qualities of tonifying yang, warming the interior, and alleviating pain and is commonly used to treat limb pain, paralysis, and cold extremities [32].

This study found that prescriptions containing Processed Aconite Root increased with the increasing ages of patients. With advancing age, the body is believed to experience more coldness, leading to pain and numbness. If correcting an abnormality still results in residual pain, adding Processed Aconite Root to the treatment regimen may effectively address the pain and numbness.

The findings of this study highlight the significant role played by Kampo medicines in the anesthesiology department, showcasing their diverse and effective use in pain management. The varied prescription patterns demonstrate the adaptability of Kampo medicines and a personalized approach. Integration of Kampo medicines with Western medicine warrants the necessity of comprehensive knowledge of these medications, and collaboration among healthcare providers is crucial. The insights from this study can guide future clinical practices and research in Kampo medicines. Future studies must explore the long-term outcomes and efficacy of Kampo formulations across different patient populations.

### Limitations

This study had several limitations. First, since this was a single-center retrospective analysis, the findings may not be generalizable to other institutions or patient populations. Second, the study relied on medical records, which may contain inaccuracies or incomplete data. Third, the observational nature of the study does not allow for causal inferences regarding the effectiveness of Kampo formulations. In addition, this study is based solely on Kampo prescriptions recorded in the electronic medical records of our hospital. Therefore, it does not include patients who may be taking Kampo medicine as over-the-counter (OTC) products or receiving Kampo prescriptions from other medical institutions. These external sources of Kampo usage may contribute to overall consumption trends but are beyond the scope of this analysis.

## Data Availability

The data that support the findings of this study are not openly available and are available from the corresponding author upon reasonable request.

## References

[1] Shibata R, Chu G-X, Lin X, Fu J-R. Development and future trends of traditional Kampo medicine in Japan. Chin Med Cult. 2022;5(1):58–64.

[2] Chu G-X, Chen L, Yan H, Ren Y, Zhu G. Historical evolution of traditional medicine in Japan. Chin Med Cult. 2019;2:36–43.

[3] Arai I. Clinical studies of traditional Japanese herbal medicines (Kampo): need for evidence by the modern scientific methodology. Integr Med Res. 2021;10(3):100722. 34136346 10.1016/j.imr.2021.100722 PMC8181179

[4] Terajima Y, Arai Y, Makino I, Ikemoto T, Saisu H, Owari K. Kampo for the treatment of pain in japan: A review. Pain Therapy. 2020;9:161–70. 32157597 10.1007/s40122-020-00160-w PMC7203354

[5] Yoshino T. The integration of traditional medicine with conventional biomedicine: A narrative review of the Japanese perspective. J Integr Complement Med. 2023;29(6–7):372–9. 36961400 10.1089/jicm.2022.0643 PMC10280171

[6] Uneda K, Yoshino T, Ito H, Imoto S, Nogami T. Current situation and future issues with Kampo medicine: A survey of Japanese physicians. Traditional Kampo Med. 2024;11(2):156–66.

[7] Saito K, Maegawa H, Nakamura T. Regulation of traditional herbal medicinal products in Japan. J Ethnopharmacol. 2014;158 Pt B:511–5. 10.1016/j.jep.2014.07.012 25043783

[8] Tsutani K, Motoo Y, Seki T. Traditional Japanese medicine, kampo: its history and current status. Chin J Integr Med. 2011;17:85–7. 21390572 10.1007/s11655-011-0653-y

[9] Takayama S, Arita R, Kikuchi A, Ohsawa M, Kaneko S, Ishii T. Clinical practice guidelines and evidence for the efficacy of traditional Japanese herbal medicine (Kampo) in treating geriatric patients. Front Nutr. 2018;5:66. 30083536 10.3389/fnut.2018.00066 PMC6064728

[10] Takayama S, Akaishi T, Nozaki H, Suzuki S, Arita R, Saito N, et al. Characteristics and course of patients treated with Kampo medicine in the department of general medicine. J Gen Family Med. 2020;21(3):48–55. 10.1002/jgf2.294 PMC7260161 32489756

[11] Takayama S, Namiki T, Arita R, Ono R, Kikuchi A, Ohsawa M, et al. Contribution of traditional Japanese Kampo medicines, Kakkonto with shosaikotokakikyosekko, in treating patients with mild-to-moderate coronavirus disease 2019: further analysis of a multicenter, randomized controlled trial. J Infect Chemother. 2023;29(11):1054–60. 37507087 10.1016/j.jiac.2023.07.013

[12] Arai I. Clinical studies of traditional Japanese herbal medicines (Kampo): need for evidence by the modern scientific methodology. Integr Med Res. 2021;10:100722. 34136346 10.1016/j.imr.2021.100722 PMC8181179

[13] World Health Organization. International Classification of Diseases 11th Revision (ICD-11): World Health Organization. 2022 [Available from https://www.who.int/standards/classifications/classification-of-diseases]

[14] World Health Organization. Frequently Asked Questions: Traditional Medicine: World Health Organization. 2025 [Available from: https://www.who.int/standards/classifications/frequently-asked-questions/traditional-medicine

[15] Aaberg C, Dahmen H, Davies C, Sandau PL, Srinivasan R. ISO 9001:2015 versus ICH Q10 - A comparison. PDA J Pharm Sci Technol. 2021 Mar-Apr;75(2):188–206. 10.5731/pdajpst.2020.011692 32999074

[16] Agency PaMD. Japanese Pharmacopoeia 18th Edition: Yakuji-Nippo, Tokyo; [Available from: Japanese Pharmacopohttps://www.pmda.go.jp/english/rs-sb-std/standards-development/jp/0029.htmleia 18th Edition.

[17] Arai Y-C, Makino I, Ikemoto T, Saisu H, Terajima Y, Owari K. Kampo for the treatment of pain in japan: A review. Pain Therapy. 2020;9(1):161–70. 32157597 10.1007/s40122-020-00160-w PMC7203354

[18] Takayama S, Kobayashi S, Kaneko S, Tabata M, Sato S, Ishikawa K, et al. Improving the quality of postgraduate education in traditional Japanese Kampo medicine for junior residents: an exploratory survey conducted in five institutions in the Tohoku area. Tohoku J Exp Med. 2016;240(3):235–42. 27890870 10.1620/tjem.240.235

[19] Yasui T, Yamada M, Uemura H, Ueno S, Numata S, Ohmori T, Tsuchiya N, Noguchi M, Yuzurihara M, Kase Y, Irahara M. Changes in Circulating cytokine levels in midlife women with psychological symptoms with selective serotonin reuptake inhibitor and Japanese traditional medicine. Maturitas. 2009;62(2):146–52. 19179025 10.1016/j.maturitas.2008.12.007

[20] Yasui T, Matsui S, Yamamoto S, Uemura H, Tsuchiya N, Noguchi M, Yuzurihara M, Kase Y, Irahara M. Effects of Japanese traditional medicines on Circulating cytokine levels in women with hot flashes. Menopause. 2011;18(1):85–92. 20647958 10.1097/gme.0b013e3181e5063c

[21] Namiki T, Hoshino T, Egashira N, Kogure T, Endo M, Homma M. A review of frequently used Kampo prescriptions part 1. Daikenchuto Traditional Kampo Med. 2022;9(3):151–79.

[22] Suzuki T, Kikuchi A, Kaneko A, Arita R, Nogami T, Saito N, et al. A review of frequently used Kampo prescriptions: part 2—Hangekobokuto. Traditional Kampo Med. 2023;10(2):103–19.

[23] Mizukami K, Asada T, Kinoshita T, Tanaka K, Sonohara K, Nakai R, Yamaguchi K, Hanyu H, Kanaya K, Takao T, Okada M, Kudo S, Kotoku H, Iwakiri M, Kurita H, Miyamura T, Kawasaki Y, Omori K, Shiozaki K, Odawara T, Suzuki T, Yamada S, Nakamura Y, Toba K. A randomized cross-over study of a traditional Japanese medicine (kampo), yokukansan, in the treatment of the behavioural and psychological symptoms of dementia. Int J Neuropsychopharmacol. 2009;12(2):191–9. 19079814 10.1017/S146114570800970X

[24] Nagata K, Yokoyama E, Yamazaki T, Takano D, Maeda T, Takahashi S, Terayama Y. Effects of Yokukansan on behavioral and psychological symptoms of vascular dementia: an open-label trial. Phytomedicine. 2012;19(6):524–8. 22421528 10.1016/j.phymed.2012.02.008

[25] Takashi. Kuwaki.Zitsuyotyuuigakunaikagaku(BOOK).Touyouigakukokusaikenkyudan 1990.

[26] Unschuld PU. Huang Di Nei Jing Su Wen: nature, knowledge, imagery in an ancient Chinese medical text. With an appendix, the doctrine of the five periods and six Qi in the Huang Di Nei Jing Su Wen. Berkeley, Calif: University of California Press; 2003. p. 521.

[27] Shu H, Arita H, Hayashida M, Zhang L, An K, Huang W, Hanaoka K. Anti-hypersensitivity effects of Shu-jing-huo-xue-tang, a Chinese herbal medicine, in CCI-neuropathic rats. J Ethnopharmacol. 2010;131(2):464–70. 20633621 10.1016/j.jep.2010.07.004

[28] Okayasu I, Tachi M, Suzue E, Ito N, Ozaki Y, Mishima G, Kurata S, Ayuse T. A case report of burning mouth syndrome with dry mouth managed by Kampo medicine. Anesth Prog. 2023;70(3):134–6. 37850679 10.2344/anpr-70-02-10 PMC11080977

[29] Suzuki H, Inadomi JM, Hibi T. Japanese herbal medicine in functional Gastrointestinal disorders. Neurogastroenterol Motil. 2009;21(7):688–96. 19563404 10.1111/j.1365-2982.2009.01290.x PMC2943019

[30] Nakatani Y, Negoro K, Yamauchi M, Katasho M, Ishikura KI, Iwaki A, Tsukada K, Yamaguchi M, Uehara A, Yoshida M, Ishiuchi K, Makino T, Kitajima M, Ohsawa M, Amano T. Neoline, an active ingredient of the processed aconite root in Goshajinkigan formulation, targets Nav1.7 to ameliorate mechanical hyperalgesia in diabetic mice. J Ethnopharmacol. 2020;259:112963. 32439405 10.1016/j.jep.2020.112963

[31] Yamaguchi H, Yoshino T, Oizumi H, Arita R, Nogami T, Takayama S. A review of frequently used Kampo prescriptions. Part 3. Yokukansan Traditional Kampo Med. 2023;10(3):197–223.

[32] Kondo T. Suppressive effects of processed aconite root on dexamethasone-induced muscle ring finger protein-1 expression and its active ingredients. J Nat Med. 2022;76(3):594–604. 35178660 10.1007/s11418-022-01606-5 PMC10008256

